# Effects of lifetime cumulative ginseng intake on cognitive function in late life

**DOI:** 10.1186/s13195-018-0380-0

**Published:** 2018-05-24

**Authors:** Silvia Kyungjin Lho, Tae Hui Kim, Kyung Phil Kwak, Kayoung Kim, Bong Jo Kim, Shin Gyeom Kim, Jeong Lan Kim, Tae Hyun Kim, Seok Woo Moon, Jae Young Park, Joon Hyuk Park, Seonjeong Byun, Seung Wan Suh, Ji Young Seo, Yoonseop So, Seung-Ho Ryu, Jong Chul Youn, Kyoung Hwan Lee, Dong Young Lee, Dong Woo Lee, Seok Bum Lee, Jung Jae Lee, Ju Ri Lee, Hyeon Jeong, Hyun-Ghang Jeong, Jin Hyeong Jhoo, Kyuhee Han, Jong Woo Hong, Ji Won Han, Ki Woong Kim

**Affiliations:** 1 Department of Neuropsychiatry, Seoul National University Bundang Hospital, 166 Gumiro, Bundanggu, Seongnamsi, Gyeonggido 463-707 Korea; 2 Department of Psychiatry, Yonsei University Wonju Severance Christian Hospital, Wonju, Gangwon-do Korea; 3 Department of Psychiatry, Dongguk University Gyeongju Hospital, Gyeongju, Korea; 4 Department of Psychiatry, National Center for Mental Health, Seoul, Korea; 5 Department of Psychiatry, Gyeongsang National University School of Medicine, Jinju, Korea; 6 Department of Neuropsychiatry, Soonchunhyang University Bucheon Hospital, Bucheon-si, Gyeonggi-do Korea; 7 Department of Psychiatry, School of Medicine, Chungnam National University, Daejeon, Korea; 8 Department of Psychiatry, School of Medicine, Konkuk University, Konkuk University Chungju Hospital, Chungju, Korea; 9 Department of Neuropsychiatry, Jeju National University Hospital, Jeju, Korea; 10 Department of Neuropsychiatry, Changwon Gyeongsang National University Hospital, Jinju, Korea; 11 Department of Psychiatry, School of Medicine, Konkuk University, Konkuk University Medical Center, Seoul, Korea; 12 Department of Neuropsychiatry, Kyunggi Provincial Hospital for the Elderly, Yongin, Korea; 13 Department of Psychiatry, Bongseng Memorial Hospital, Busan, Korea; 14 Department of Neuropsychiatry, Seoul National University Hospital, Seoul, Korea; 15 Department of Psychiatry, Seoul National University College of Medicine, Seoul, Korea; 16 Department of Neuropsychiatry, Inje University Sanggye Paik Hospital, Seoul, Korea; 17 Department of Psychiatry, Dankook University Hospital, Cheonan, Chungcheongnam-do Korea; 18 Department of Psychiatry, Korea University Guro Hospital, Korea University College of Medicine, Seoul, Korea; 19 Department of Psychiatry, Kangwon National University, School of Medicine, Chuncheon, Korea; 20 Department of Brain and Cognitive Science, Seoul National University College of Natural Sciences, Gwanak-gu, Seoul, Korea

**Keywords:** Ginseng, Panax, Aged, Cognition, Cohort studies, Longitudinal studies

## Abstract

**Background:**

We investigated the effects of lifetime cumulative ginseng intake on cognitive function in a community-dwelling population-based prospective cohort of Korean elders.

**Methods:**

Community-dwelling elders (*N* = 6422; mean age = 70.2 ± 6.9 years, education = 8.0 ± 5.3 years, female = 56.8%) from the Korean Longitudinal Study on Cognitive Aging and Dementia were included. Among them, 3918 participants (61.0%) completed the 2-year and 4-year follow-up evaluations. Subjects were categorized according to cumulative ginseng intake at baseline evaluation; no use group, low use (< 5 years) group, and high use (≥ 5 years) group. One-way analysis of covariance (ANCOVA) was conducted to compare the impact of cumulative ginseng intake on baseline Consortium to Establish a Registry for Alzheimer’s Disease Assessment Packet neuropsychological battery total score (CERAD total score) and Mini-Mental State Examination (MMSE) score among the three groups while adjusting for potential covariates. A repeated-measures ANCOVA was performed to investigate the impacts on the changes in CERAD total scores and MMSE scores during the 4 years of follow-up.

**Results:**

The high use group showed higher CERAD total scores compared to the no use group after controlling for age, sex, education years, socioeconomic status, smoking, alcohol intake, presence of hypertension, stroke history, Geriatric Depression Scale, Cumulative Illness Rating Scale, and presence of the APOE e4 allele (*F*(2, 4762) = 3.978, *p* = 0.019). The changes of CERAD total score for 2 or 4 years of follow-up did not differ according to the use of ginseng.

**Conclusions:**

Cumulative ginseng use for longer than 5 years may be beneficial to cognitive function in late life.

**Electronic supplementary material:**

The online version of this article (10.1186/s13195-018-0380-0) contains supplementary material, which is available to authorized users.

## Background

Ginseng, which has been used for at least 2000 years in Asian countries [1], is one of the most widely sold medicinal herbs worldwide [2]. The estimated world ginseng market is dramatically increasing, worth approximately $2085 million in 2009 [1]. An analysis of the Korean Ministry of Food and Drug Safety in the year 2015 reported that ginseng products, including red ginseng and white ginseng, reached KRW 725.0 billion, with the highest market share reaching 39.8% of the dietary supplement market in South Korea.

Ginseng is popular because it is effective in boosting immune function, it has antifatigue effects, and it improves cognitive functions [3]. Ginseng has the potential to slow cognitive decline in elderly individuals or reduce the risk of dementia, which is of great scientific and public health interest. Biological data suggest that various functional constituents, especially ginseng saponins (also known as ginsenosides), improve brain cholinergic function, decrease inflammation, and reduce production of amyloid beta proteins that directly limit the progression of Alzheimer’s disease (AD) pathology [4–7].

Recently, four randomized control trials (RCTs) [8–11] of patients with AD revealed that red ginseng might be effective for cognitive improvement. Among the four RCTs, one study reported that the cognitive functions of AD patients improved and were maintained with both 4.5 g/day and 9.0 g/day of red ginseng supplements during 2 years of follow-up [10]. However, a systematic review and meta-analysis [12] based on these four RCTs concluded that the findings regarding the effects of ginseng on AD were inconclusive due to the limitations of these studies, which included small sample sizes and lack of placebo control design. In terms of participants without dementia, a systematic review published in 2010 [2], which was based on the results of nine double-blind RCTs, showed that there is insufficient evidence regarding the efficacy of ginseng on cognitive function due to limited sample sizes and only short-term follow-up (up to 12 weeks).

Due to their inherent design characteristics, RCTs cannot sufficiently reflect long-term ginseng intake; therefore, the effects of long-term ginseng intake on cognition should be studied in a longitudinal prospective cohort. To our knowledge, the only instance of such a study is the one by Persson et al. [13], a prospective cohort study in Sweden that reported no association between long-term ginseng use and cognitive performance. However, the participants in that study were aged 35–80 years, therefore making it difficult to assess the effects of ginseng on the elderly population. Additionally, the study was conducted in a western country, where ginseng intake is generally lower than it is in Asian countries. Among 3500 participants, 86 subjects (2.5%) had previously used ginseng; therefore, if the effect size was small, the effect of ginseng would be difficult to observe. The study focused on revealing the relationship between long-term ginseng intake and memory function, but there was no follow-up evaluation showing the trajectory of cognitive performance over time.

There is still little research regarding how ginseng usage correlates with cognitive function in elderly populations with and without cognitive impairment, and we also have little information on the longitudinal effects of ginseng. Furthermore, to our knowledge, a large population cohort has never been studied in Asia, especially not in Korea, where the amount of ginseng distribution is the highest worldwide [1].

In this study, we investigated the correlation between lifetime cumulative use of ginseng and cognitive function in a large, community-dwelling, population-based prospective cohort of elders in Asia, especially in Korea. In addition, we sought to determine the effect of cumulative ginseng intake on cognitive function during a follow-up period of 4 years.

## Methods

### Subjects

**Fig. 1:**
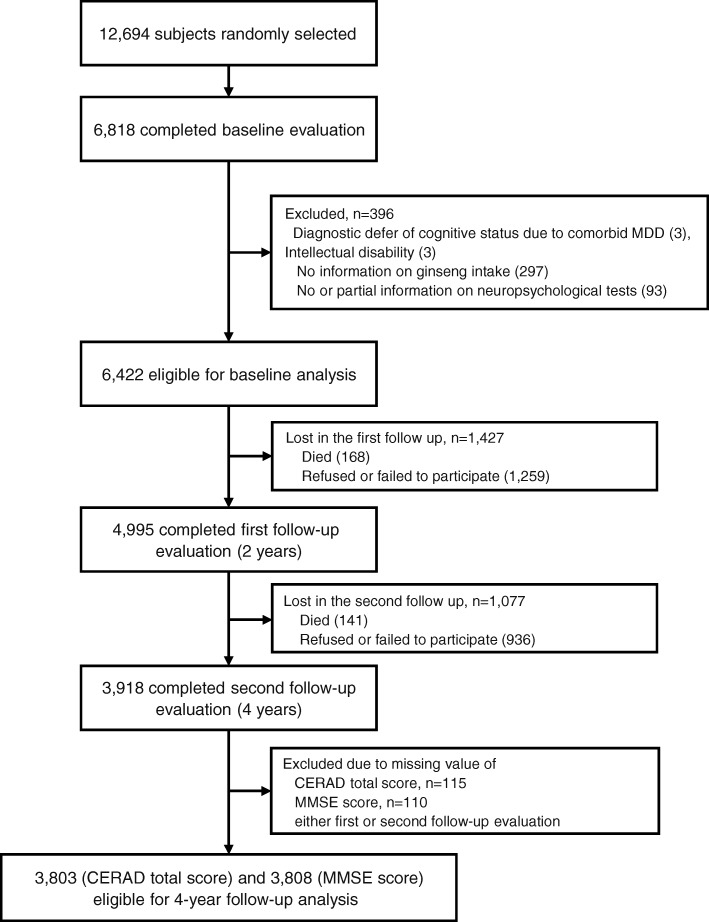
Enrollment and follow-up of study participants. CERAD Consortium to Establish a Registry for Alzheimer’s Disease, MDD major depressive disorder, MMSE Mini-Mental State Examination

This study was conducted as a part of the Korean Longitudinal Study on Cognitive Aging and Dementia (KLOSCAD), which is a population-based prospective cohort study of Korean elders aged 60 years and older [14]. Among the random sample (*N* = 12,694) drawn from Koreans, 6818 participants completed an evaluation of cognitive function during the baseline study period, between 2010 and 2012 (response rate = 53.7%). Participants with partial or no information regarding neuropsychological tests, with a diagnostic deferral due to comorbid major depressive disorder or intellectual disability, or with no information regarding ginseng intake were excluded (*N* = 396; Fig. 1). As a result, 6422 participants (mean age = 70.2 ± 6.9 years, education = 8.0 ± 5.3 years, female = 56.8%) were eligible for baseline analyses. Follow-up evaluations were conducted every 2 years, and the present study included the first and second follow-up studies, obtained during 2012–2014 and 2014–2016, respectively. Of the 6422 subjects included in this study, 3918 participants (61.0%) completed the first and second follow-up evaluations. All participants were fully informed of the study protocol and provided written informed consent, signed by the subjects themselves or their legal guardians. The study protocol was approved by the Institutional Review Board of the Seoul National University Bundang Hospital.

### Cumulative ginseng intake

At each evaluation, the age at initial ginseng intake and the number of days in a month of ginseng intake (i.e., (1) white ginseng or (2) red ginseng in any formula containing ginseng extract) were recorded using a questionnaire. Because the amount of ginseng varies considerably between ginseng products and there is a low probability of obtaining the exact amount of ginseng in any given product, we defined “cumulative ginseng intake” by subtracting the age at initial ginseng intake from the age at study entry, adding 1 year to reflect the intake of the first year, and then multiplying the result by a year’s value of intake, calculated by multiplying the number of days of ginseng intake in a month by 12 months. As the median of the years of cumulative ginseng intake in participants with ginseng consumption was 5 years, ginseng use was categorized as “no use,” “low use” (< 5 years), or “high use” (≥ 5 years).

### Diagnostic assessments of cognitive disorders and comorbid conditions

Geriatric neuropsychiatrists with expertise in dementia research administered a face-to-face standardized diagnostic interview as well as physical and neurological examinations, using the Korean version of the Consortium to Establish a Registry for Alzheimer’s Disease Assessment Packet Clinical Assessment Battery (CERAD-K-C) [15] to diagnose cognitive disorders. Comorbid mental disorders, including depressive disorders, were evaluated using the Korean version of the Mini International Neuropsychiatric Interview (MINI-K) [16, 17]. Research neuropsychologists or trained research nurses administered the Korean version of the Consortium to Establish a Registry for Alzheimer’s Disease Assessment Packet Neuropsychological Assessment Battery (CERAD-K-N) [15, 18]. The CERAD-K-N consists of nine neuropsychological tests: (1) the Verbal Fluency Test, (2) the 15-item Boston Naming Test, (3) the Mini Mental Status Examination for dementia screening (MMSE-DS) [19], (4) the Word List Memory Test, (5) the Constructional Praxis Test, (6) the Word List Recall Test, (7) the Word List Recognition Test, (8) the Constructional Recall Test, and (9) the Trail Making Test A/B [15, 18]. The CERAD total score was calculated by summation of CERAD-K-N subtest scores, excluding the MMSE score [20].

Dementia was diagnosed according to the diagnostic criteria in the *Diagnostic and Statistical Manual of Mental Disorders*, fourth edition (DSM-IV) [21]. Mild cognitive impairment (MCI) was diagnosed according to the Consensus Criteria from the International Working Group on MCI [22]. The presence of objective cognitive impairment was ascertained when the performance of the subjects was − 1.5 standard deviations (SDs) or below the mean age, gender, and education-adjusted values in any of neuropsychological tests.

### Sociodemographic factors, lifestyle factors, and comorbid illnesses

Sociodemographic factors included age, sex, years of education, and socioeconomic status (SES). The SES was defined using medical insurance status, which was categorized into two groups: medical insurance group and Medicaid/Medicare group. Duration of smoking (years), amount of smoking per day (in order to calculate number of pack-years), and amount of alcohol consumption in units over the lifetime were recorded. Body mass index (BMI) was calculated by dividing weight by the square of height (expressed in kg/m^2^). Presence of hypertension or history of stroke was identified by self-report or electronic database, and the burden of comorbid illnesses was identified using the Cumulative Illness Rating Scale (CIRS) [23]. Depressive symptom severity was assessed using the Korean version of the Geriatric Depression Scale (GDS-K) [24].

### Statistical analyses

A one-way analysis of variance (ANOVA) for continuous variables and a linear-by-linear test for categorical variables were used to compare the baseline sociodemographic and clinical characteristics between the no use, low use, and high use groups for cumulative ginseng intake. Post-hoc analysis using the Bonferroni method was performed. A one-way analysis of covariance (ANCOVA) was conducted in order to compare the impact of cumulative ginseng intake on baseline CERAD total and MMSE scores across the three groups (no use, low use, and high use) while adjusting for age, sex, years of education, SES, smoking (pack-years), alcohol intake (units in lifetime), presence of hypertension, stroke history, GDS-K, CIRS, and presence of APOE e4 allele. Ordinal logistic regression using the generalized linear model was conducted in order to compare the impact of cumulative ginseng intake on the baseline clinical diagnosis while adjusting for the same confounding variables as those of the ANCOVA model.

To investigate of the impacts of cumulative ginseng intake on changes in CERAD total and MMSE scores from baseline to the first (2 years) and second (4 years) follow-up evaluations, repeated-measures ANCOVAs were performed with time and group as independent factors and age, sex, years of education, SES, smoking (pack-years), alcohol intake (units in lifetime), presence of hypertension, stroke history, GDS-K, CIRS, presence of the APOE e4 allele, and ginseng intake amount during 4 years as covariates. Analyses were performed with SPSS software (version 23.0; SPSS, Chicago, IL, USA).

## Results

### Baseline characteristics according to cumulative ginseng intake

At baseline evaluation, among 6422 participants, 677 subjects (10.6%) had ever used ginseng. The low use group (below median, < 5 years) included 491 subjects, and the high use group (above median, ≥ 5 years) included 186 subjects (Table 1). The mean age of the no use group (70.4 ± 7.0 years) was significantly higher than that of the low use group (68.6 ± 6.0 years; *F* = 16.3, *p* < 0.001). Men (11.3%) were more likely to take ginseng than women (10.0%). Subjects with higher use (i.e., low use group vs no use group, high use group vs low use group) showed lower percentages of women (*p* < 0.001), had more years of education (*F* = 85.185, *p* < 0.001), and were more likely to be in a higher SES group (*p* < 0.001). The BMI of the no use group (24.1 ± 3.1) was significantly higher than that of the low use group (23.7 ± 2.9; *F* = 5.138, *p* = 0.006). The group with no use of ginseng showed higher GDS-K scores (10.2 ± 6.6) than that of the low use group (9.3 ± 6.3) and high use group (8.5 ± 5.9; *F* = 8.770, p < 0.001). Subjects in the high use group had higher CIRS scores (5.2 ± 3.0) than those of the no use group (4.5 ± 2.8) and low use group (4.8 ± 2.8; *F* = 7.453, *p* = 0.001).

**Table 1:** Sociodemographic and clinical characteristics among groups divided according to cumulative ginseng intake

Characteristic		Cumulative ginseng intake				
	Total (*N* = 6422)	No use group (*N* = 5745)	Low use group (< 5 years) (*N* = 491)	High use group (≥ 5 years) (*N* = 186)	*p* value^a^	Post hoc^b^
Cumulative ginseng intake (years)	0.46 ± 2.28	0.0	1.85 ± 1.26	11.0 ± 7.21	< 0.001	0 < 1 < 2
Cumulative ginseng intake (amounts)	166.3 ± 820.8	0.0	663.4 ± 404.2	3989.7 ± 2595.7	< 0.001	0 < 1 < 2
Age (years)	70.2 ± 6.92	70.4 ± 7.0	68.6 ± 6.0	69.7 ± 5.9	< 0.001	0 > 1
Female, *n* (%)	3649 (56.8)	3316 (57.7)	252 (51.3)	81 (43.5)	< 0.001	
Education (years)	8.0 ± 5.3	7.7 ± 5.3	9.7 ± 5.1	11.9 ± 4.8	< 0.001	0 < 1 < 2
SES (medical insurance), *n* (%)	6186 (95.0)	5529 (94.8)	472 (95.4)	185 (98.9)	0.024	
BMI (kg/m^2^)^c^	24.1 ± 3.1	24.1 ± 3.1	23.7 ± 2.9	23.7 ± 2.7	0.006	0 > 1
Smoking (pack-years)^c^	10.5 ± 29.8	10.7 ± 30.9	9.6 ± 18.2	8.9 ± 16.7	0.575	
Alcohol intake (units in lifetime)^c^	1.3 × 10^4^ ± 4.2 × 10^4^	1.3 × 10^4^ ± 4.3 × 10^4^	1.3 × 10^4^ ± 3.1 × 10^4^	1.5 × 10^4^ ± 3.1 × 10^4^	0.768	
Presence of hypertension, *n* (%)	3399 (52.9)	3046 (53.0)	252 (51.3)	101 (54.3)	0.869	
Stroke history, *n* (%)	508 (7.9)	463 (8.1)	34 (6.9)	11 (5.9)	0.174	
APOE e4 carrier, *n* (%)^c^	1215 (23.3)	1058 (22.9)	119 (27.9)	38 (22.8)	0.186	
GDS-K^c^	10.1 ± 6.6	10.2 ± 6.6	9.3 ± 6.3	8.5 ± 5.9	< 0.001	0 > 1, 0 > 2
CIRS^c^	4.5 ± 2.8	4.5 ± 2.8	4.8 ± 2.8	5.2 ± 3.0	0.001	0 < 2

Data shown as mean ± standard deviation for continuous variables

*BMI* body mass index, *CIRS* cumulative illness rating scale, *GDS-K* Korean version of the Geriatric Depression Scale, *SES* socioeconomic status

^a^Derived from one-way analysis of variance for continuous variables, from a linear-by-linear association test for categorical variables

^b^Post-hoc analysis using Bonferroni; 0, 1, and 2 denote nonusers, low ginseng intake group (< 5 years), and high ginseng intake group (≥ 5 years) respectively

^c^Missing values: BMI, *n* = 482; smoking, *n* = 62; alcohol intake, *n* = 52; APOE e4 carrier, *n* = 1212; GDS-K, *n* = 237; CIRS, *n* = 2

There were no differences in smoking habits, alcohol intake, rate of hypertension, stroke history, and presence of the APOE e4 allele relative to lifetime cumulative ginseng intake.

### Impact of cumulative ginseng intake on the baseline neuropsychological performance

The low use group showed higher CERAD total scores than those of the no use group, and the high use group showed better scores than the others (*F* = 47.049, *p* < 0.001; Table 2). The low use and high use groups showed higher MMSE-DS scores than the no use group (*F* = 32.825, *p* < 0.001). There was a significant effect of cumulative ginseng intake on CERAD total score after controlling for covariates, including age, sex, years of education, SES, smoking, alcohol intake, presence of hypertension, stroke history, GDS-K, CIRS, and presence of the APOE e4 allele (*F*(2, 4762) = 3.978, *p* = 0.019). A post hoc test showed that the high use group had higher CERAD total scores than the no use group (*p* = 0.018). However, there was no difference in MMSE-DS scores between the groups after adjusting for the covariates mentioned earlier.

**Table 2:** Impact of cumulative ginseng intake on baseline CERAD total and MMSE-DS scores

	Cumulative ginseng intake			*F* value for ANOVA *F*(2, 6420)	Post hoc^a^	*F* value for ANCOVA^b^ *F*(2, 4762)	Post hoc^a^
Baseline neuropsychological tests	No use (*N* = 5745)	Low use (< 5 years) (*N* = 491)	High use (≥ 5 years) (*N* = 186)				
CERAD total score	60.2 ± 14.9	64.8 ± 12.3	68.1 ± 10.8	47.049^*^	0 < 1 < 2		
	62.3 ± 13.6	65.9 ± 11.7	68.5 ± 10.7			3.978^**^	0 < 2
MMSE-DS score	25.2 ± 4.2	26.3 ± 3.1	27.0 ± 2.2	32.825^*^	0 < 1, 0 < 2		
	25.9 ± 0.043	26.1 ± 0.139	26.2 ± 0.221			1.174	

Data shown as mean ± standard deviation for analysis of variance (ANOVA), adjusted mean ± standard error for analysis of covariance (ANCOVA)

*CERAD* Consortium to Establish a Registry for Alzheimer’s Disease, *MMSE-DS* Mini-Mental State Examination for dementia screening

^*^p < 0.001; ^**^p = 0.019

^a^Post-hoc analysis using Bonferroni; 0, 1, and 2 denote no-users, low ginseng intake group (< 5 years), and high ginseng intake group (≥ 5 years) respectively

^b^ANCOVA adjusted for age, sex, years of education, socioeconomic status, body mass index, smoking (pack-years), alcohol intake (units in lifetime), presence of hypertension, stroke history, Korean version of Geriatric Depression Scale, Cumulative Illness Rating Scale, and presence of APOE e4 allele

### Impact of cumulative ginseng intake on the baseline clinical diagnosis

Percentage of cognitive impairment (MCI or dementia) was significantly higher in the low use group (27.1%) than in the high use group (24.7%), and was also higher in the no use group (32.6%) than in the other groups (*p* < 0.001; Table 3). However, after controlling for potential covariates which might have influenced baseline clinical diagnosis, low use or high use of cumulative ginseng intake did not decrease the ordered log odds of being in a higher level of diagnosis (higher level, normal cognition (NC) < MCI < dementia; high use, *B* = − 0.201, standard error = 0.1993, *p* = 0.312; low use, *B* = − 0.032, standard error = 0.1247, *p* = 0.800; Table 3).

**Table 3:** Impact of cumulative ginseng intake on baseline clinical diagnosis

	Baseline clinical diagnosis, *n* (%)			*p* value^a^	Ordinal logistic regression			*p* value^b^
	NC	MCI	Dementia		*B*	SE	95% CI	
Ginseng				< 0.001				
No use	3870 (67.4)	1610 (28.0)	265 (4.6)		(Reference)			
Low use (< 5 years)	358 (72.9)	124 (25.3)	9 (1.8)		−0.032	0.1247	(− 0.276, 0.213)	0.800
High use (≥ 5 years)	140 (75.3)	45 (24.2)	1 (0.5)		−0.201	0.1993	(− 0.592, 0.189)	0.312
Total	4368 (68.0)	1779 (27.7)	275 (4.3)					

*CI* confidence interval, *MCI* mild cognitive impairment, *NC* normal cognition, *SE* standard error

^a^Derived from a linear-by-linear association test

^b^Derived from ordinal logistic regression using generalized linear model adjusted for age, sex, years of education, socioeconomic status, body mass index, smoking (pack-years), alcohol intake (units in lifetime), presence of hypertension, stroke history, Korean version of Geriatric Depression Scale, Cumulative Illness Rating Scale, and presence of APOE e4 allele

**Table 4:** Impact of cumulative ginseng intake on cognitive trajectory over 4 years

	CERAD total score (*N* = 3084)^a^			*F* value	*p* value^b^	MMSE-DS (*N* = 3080)^c^			*F* value	*p* value^b^
	Baseline	2-year	4-year			Baseline	2-year	4-year		
Ginseng				1.062	0.346				0.223	0.800
No use	64.9 ± 12.2	65.8 ± 13.4	66.1 ± 14.3			26.3 ± 3.1	26.2 ± 3.4	26.3 ± 3.5		
Low use (< 5 years)	68.0 ± 10.9	69.4 ± 11.6	69.5 ± 12.0			27.0 ± 2.4	27.0 ± 2.5	27.0 ± 3.0		
High use (≥ 5 years)	70.3 ± 10.3	70.9 ± 10.3	70.5 ± 11.0			27.2 ± 1.8	27.5 ± 2.0	27.1 ± 2.2		
time				58.002	< 0.001				20.165	< 0.001
Group × time				1.653	0.159				0.667	0.613

Data shown as mean ± standard deviation

*CERAD* Consortium to Establish a Registry for Alzheimer’s disease, *MMSE-DS* Mini-Mental State Examination for dementia screening

^a^719 from 3803 subjects excluded due to missing values of covariates

^b^*p* value in repeated-measures analysis of covariance model adjusting for age, sex, years of education, socioeconomic status, body mass index, smoking (pack-years), alcohol intake (units for lifetime), presence of hypertension, stroke history, Korean version of geriatric depression scale, cumulative illness rating scale, presence of APOE e4 allele, and ginseng intake amount during 4 years

^c^728 from 3808 subjects excluded due to missing values of covariates

### Impact of cumulative ginseng intake on cognitive trajectory

Among the 6422 participants, 3918 subjects (61.0%) completed both the first and second follow-up evaluations (Fig. 1). During the first follow-up study, 168 participants (2.6%) died and 1259 participants (19.6%) refused or failed to participate in the follow-up for either an unknown reason or institutionalization. Excluding the 1427 participants (22.2%) lost in the first follow-up study, 1077 subjects (16.8%) were additionally lost during the second follow-up period because 141 (2.2%) died and 936 (14.6%) refused or failed to participate in the second follow-up. Among the 3918 participants who completed the first and second follow-up evaluations, participants with missing CERAD total scores and MMSE-DS scores at either first or second follow-up evaluations were excluded, leaving 3803 and 3808 participants for use in a repeated-measures ANOVA (RMANOVA) of CERAD total scores and MMSE-DS scores, respectively.

**Fig. 2:**
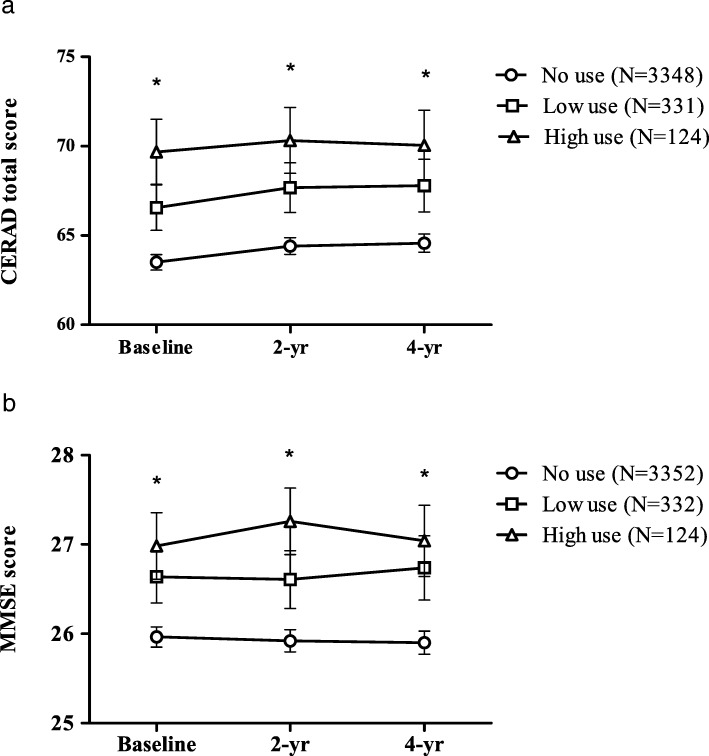
Change in (**a**) CERAD total score and (**b**) MMSE-DS score at baseline, 2 years, and 4 years. ^*^p < 0.01 for group effect at each time point in model for repeated-measures ANOVA. CERAD Consortium to Establish a Registry for Alzheimer’s Disease, MMSE Mini-Mental State Examination, yr year

There was significant group effect (*F* = 19.974, *p* < 0.001) and time effect (*F* = 7.505, *p* = 0.001) in the repeated-measures ANOVA for the CERAD total score, but no significant time and group interaction (*F* = 0.361, *p* = 0.837). For the MMSE score, the group effect was significant (*F* = 14.526, *p* < 0.001), but there was neither significant time effect (*F* = 0.304, *p* = 0.738) nor time and group interaction (*F* = 0.951, *p* = 0.433; Fig. 2).

After controlling for age, sex, years of education, SES, smoking, alcohol intake, presence of hypertension, stroke history, GDS-K, CIRS, presence of APOE e4 allele, and amount of ginseng intake during 4 years in the repeated-measures ANCOVA model, there was no group effect for CERAD total score and MMSE score (*F* = 1.060, df = 2.0, *p* = 0.346 for CERAD total score; *F* = 0.220, df = 2.0, *p* = 0.800 for MMSE). Although significant time effects were shown for both CERAD total score (*F* = 58.002, *p* < 0.001) and MMSE-DS score (*F* = 20.165, *p* < 0.001), there was no significant time and group interaction for either CERAD total score (*F* = 1.653, *p* = 0.159) or MMSE-DS score (*F* = 0.667, *p* = 0.613; Table 4).

## Discussion

The purpose of this study was to determine whether or not lifetime cumulative ginseng intake has beneficial effects on cognitive function and future change. Here, we found that individuals with high lifetime cumulative ginseng intake showed higher CERAD total scores in late life than nonusers, even after comprehensively controlling for confounding variables that could affect cognitive function. However, changes in cognitive function over 2 or 4 years in late life were not influenced by the use of ginseng. To our knowledge, this study is the first that showed the effect of ginseng on late life cognitive function in a large, randomly sampled, community-based elderly cohort.

These results indicate that long-term use of ginseng may be beneficial to late life cognitive function.

The beneficial effect of ginseng on cognitive function was dose dependent. The individuals who had taken ginseng for more than 5 years showed better cognitive function than those who never took ginseng, whereas those who had taken ginseng for less than 5 years did not. There was no previous study that delineated the duration of intake that has efficacy on cognitive function in elderly populations. Many of the previous RCTs [25–27] of ginseng effect on cognitive performance were conducted with healthy volunteers who were young or middle-aged, and duration of intake ranged from days to several weeks in either 200 mg or 400 mg amounts. They suggested that ginseng might improve some aspect of cognitive function; however, the short duration of these studies and the inclusion of young subjects limited the interpretations of results regarding whether or not ginseng has longlasting effects on cognition, even with a short duration of intake in aged groups. Of course, some RCTs have also shown that high-dose (at least 4.5 g/day or 9.0 g/day), long-term (for 6 months or 2 years) ginseng intake in AD patients has an effect on cognitive function [10, 11]. However, ginseng dietary supplements do not contain such a high dose. Various commercialized ginseng products contain at most 1 g/day. There has been only one study exploring the effect of ginseng intake based on a prospective cohort study that reflects real-world settings. In the study by Persson et al. [13], which compared 86 ginseng users (including 51 participants who used ginseng for over 2 years and 35 participants who used ginseng for 5.6 months on average) and nonusers, no group difference was observed, which is consistent with our findings that the low use group (mean duration of cumulative ginseng intake 1.85 ± 1.26 years) did not show any significant difference in CERAD total score compared to the no use group.

A beneficial effect of ginseng on cognitive function was not observed in the 4-year prospective observation. The cognitive changes over 4 years were comparable between the subjects who had taken ginseng for 5 years or more at baseline and those who had never taken ginseng. This was the case when we additionally accounted for the use of ginseng during the follow-up period in the analysis. This discrepancy between the cross-sectional and prospective analyses may be attributable to several causes. First, the follow-up period may be too short to show the beneficial effects of ginseng on cognitive function. Second, the subjects who responded to the follow-up evaluations had better cognitive function at baseline than those who did not and thus were less likely to show cognitive decline over a short period. The data are presented in detail in Additional file 1: Table S1. Therefore, it may be beneficial to follow the subjects for a longer period of time.

It is not yet well understood how ginseng improves cognitive reserve (or brain reserve) [28]. Some previous studies showed that ginsenosides were protective against AD pathologies: antioxidative effects, inhibition of Aβ-induced cytotoxicity and tau phosphorylation, immunomodulatory activities on intracellular signaling pathway, cell apoptosis, and mitochondrial function [5, 6]. These neuroprotective and compensatory effects of ginseng may directly and indirectly contribute to cognitive reserve, resulting in better baseline CERAD total score in individuals who consumed ginseng over 5 years.

Although there were less cognitive disorders (i.e., MCI or dementia) in the subjects who had taken more ginseng in their lifetime at baseline, this association was not statistically significant when confounding factors were adjusted. This discrepant association of lifetime ginseng intake with cognitive function and cognitive disorders may be attributable to several factors. First, the effect of ginseng may improve cognitive function but not prevent or delay cognitive disorders. Second, the statistical power for testing the association between ginseng intake and cognitive disorders was smaller than that between ginseng intake and cognitive function in the current study. Third, subjective cognitive complaints are required to diagnose MCI in addition to objective cognitive impairments [22]. Subjective concerns on objective cognitive impairments may be different between the groups classified by the level of ginseng intake.

There are several limitations to this study. First, this study is subject to recall biases since the use of ginseng was evaluated using a questionnaire. Second, the dose effect was not analyzed using the amount of ginseng use, but was analyzed using the duration of ginseng use. Ginseng products on the market include varying amounts of ginseng as an active component (even though various commercialized ginseng products provide at most 1 g/day), which makes it difficult to measure the actual amount of ginseng intake. Third, we did not adjust for other dietary supplements that could also influence cognitive function. Lastly, the beneficial effect of ginseng on cognitive function was shown in the CERAD total score but not in the MMSE score. Compared to the CERAD Assessment Battery, the MMSE may be less sensitive to subtle cognitive changes and more susceptible to the ceiling effect in highly educated, cognitive normal people [29, 30]. In this study, 6.7% of the participants achieved a perfect score (30 points) and 35.1% scored 28 points or higher in the MMSE, while no one achieved a perfect score (100 points) and only 0.3% scored 91 points or higher in CERAD total score.

## Conclusions

Cumulative ginseng use for longer than 5 years may be beneficial for cognitive function in late life. However, its effect on the rate of cognitive decline over 4 years in late life was not observed, which warrants future studies with longer duration of follow-up.

## Additional file

Additional file 1: **Table S1.** Baseline characteristics of participants and those lost to follow-up (DOCX 22 kb)

## Abbreviations

AD: Alzheimer’s disease
ANCOVA: One-way analysis of covariance
ANOVA: One-way analysis of variance
BMI: Body mass index
CERAD total score: Consortium to Establish a Registry for Alzheimer’s Disease Assessment Packet neuropsychological battery total score
CERAD-K-C: Consortium to Establish a Registry for Alzheimer’s Disease Assessment Packet Clinical Assessment Battery
CERAD-K-N: Consortium to Establish a Registry for Alzheimer’s Disease Assessment Packet Neuropsychological Assessment Battery
CIRS: Cumulative Illness Rating Scale
DSM-IV: *Diagnostic and Statistical Manual of Mental Disorders*, fourth edition
GDS-K: Korean version of the Geriatric Depression Scale
KLOSCAD: Korean Longitudinal Study on Cognitive Aging and Dementia
MCI: Mild cognitive impairment
MMSE: Mini-Mental State Examination
MMSE-DS: Mini-Mental State Examination for dementia screening
NC: Normal cognition
RCT: Randomized control trial
SD: Standard deviation
