# Supplementary material for: Effects of lifetime cumulative ginseng intake on cognitive function in late life
Source: Alzheimers Res Ther. 2018 May 24;10:50. doi: 10.1186/s13195-018-0380-0 (PMC5968575; doi:10.1186/s13195-018-0380-0)
Supplement: Supplementary file 1 — Table S1. Baseline characteristics of participants and those lost to follow-up (DOCX 22 kb) [file 13195_2018_380_MOESM1_ESM.docx]

Table S1. Baseline characteristics of participants and those lost to follow-up

|  | Participants who completed study (N=3,918) | Lost to follow-up (N=2,504) | P value^*^ |
| --- | --- | --- | --- |
| Ginseng cumulative intake | 180.7 ± 827.9 | 143.7 ± 809.2 | 0.076 |
| Age (year) | 69.4 ± 6.4 | 71.5 ± 7.5 | <0.001 |
| Female (n, %) | 2248 (57.4) | 1401 (56.0) | 0.261 |
| Education (years) | 8.5 ± 5.3 | 7.2 ± 5.2 | <0.001 |
| SES, medical insurance (n, %) | 174 (4.4) | 144 (5.8) | 0.018 |
| BMI (kg/m^2^)^a^ | 24.1 ± 3.0 | 23.9 ± 3.1 | 0.012 |
| Smoking (pack-years)^a^ | 10.1 ± 30.6 | 11.2 ± 28.3 | 0.148 |
| Alcohol intake (units in lifetime)^a^ | 1.3×10^4^ ± 4.4×10^4^ | 1.2×10^4^ ± 3.8×10^4^ | 0.455 |
| Presence of hypertension (n, %) | 2008 (51.3) | 1391 (55.6) | 0.001 |
| Stroke history (n, %) | 275 (7.0) | 233 (9.3) | 0.001 |
| APOE e4 carrier (n, %)^a^ | 787 (23.7) | 428 (23.2) | 0.902 |
| GDS-K^a^ | 9.9 ± 6.5 | 10.3 ± 6.7 | 0.069 |
| CIRS^a^ | 4.5 ± 2.8 | 4.5 ± 2.9 | 0.810 |
| Baseline CERAD total score | 63.3 ± 13.4 | 56.8 ± 15.7 | <0.001 |
| Baseline MMSE | 25.9 ± 3.5 | 24.5 ± 4.7 | <0.001 |
| Baseline diagnosis (n, %) |  |  |  |
| NC | 2809 (71.7) | 1559 (62.3) | <0.001 |
| MCI | 1002 (25.6) | 777 (31.0) |  |
| Dementia | 107 (2.7) | 168 (6.7) |  |

Data are shown as mean ± standard deviation for continuous variables.

^*^derived from independent t-test for continuous variables and chi-square test or linear-by-linear association test for categorical variables

^a^Missing values, n; BMI, 482; smoking, 62; alcohol intake, 52; APOE e4 carrier, 1212; GDS-K, 237; CIRS, 2

BMI, body mass index; CERAD, Consortium to Establish a Registry for Alzheimer’s Disease; CIRS, cumulative illness rating scale; GDS-K, Korean version of Geriatric Depression Scale; NC, normal cognition; MCI, mild cognitive impairment; MMSE-DS, Mini-Mental State Examination for dementia screening; SES, socioeconomic status
